# The minimal clinically important difference (MCID) of the Olerud Molander Ankle Score (OMAS) in patients with unstable ankle fracture

**DOI:** 10.1007/s00402-022-04533-y

**Published:** 2022-07-11

**Authors:** Diederick Penning, Suzanne Kleipool, Susan van Dieren, Siem M. Dingemans, RODEO Collaborator Group, Elvira R. Flikweert, Bart A. van Dijkman, Jochem M. Hoogendoorn, Markus J. Parkkinen, Gert R. Roukema, J. Carel. Goslings, Nico L. Sosef, Jasper Winkelhagen, Tim Schepers

**Affiliations:** 1 Trauma Unit, Amsterdam University Medical Center, Location Meibergdreef, Amsterdam Movement Sciences, Meibergdreef 9, 1105 AZ Amsterdam, The Netherlands; 2 Department of Trauma Surgery, OLVG, Amsterdam, The Netherlands; 3 Department of Clinical Epidemiology, Amsterdam University Medical Center, Location Meibergdreef, Amsterdam, The Netherlands

**Keywords:** Ankle fracture, Measurement properties, Minimal clinical important difference, Olerud Molander ankle score, Patient-reported outcomes

## Abstract

**Introduction:**

The Olerud Molander Ankle Score (OMAS) is a widely used validated Patient Reported Outcome Measure (PROM). For clinical research, it is important to determine the Minimal Clinically Important Difference (MCID). The objective of this study was to determine the MCID of the OMAS at several moments in the follow-up, in a cohort of patients that underwent open reduction and internal fixation of unstable ankle fractures with syndesmotic injury.

**Materials and methods:**

Data for this descriptive study were extracted from a prospective randomized controlled trial, the RODEO trial. The Dutch version of the OMAS was completed at 3, 6 and 12-month follow-up and estimated at baseline. The used anchor-based methods were: mean change and ROC curve. The distribution-based methods were: 0.5SD and minimal detectable change (MDC).

**Results:**

This cohort included 148 patients. The mean OMAS score in the group with minimal improvement between 3 and 6 months was 15.0 (SD 17.5, 95%CI 9.4–20.6) and between 6 and 12 months 9.5 (SD 17.1, 95% CI 3.1–15.9). The ROC curve between 3 and 6 months resulted in a MCID of 12.5 (AUC 0.72) and between 6 and 12 months, the MCID was 7.5 (AUC 0.78). Using 0.5 SD, the MCID was 10.52 (SD 21.04) at 3 months, 11.37 (SD 22.73) at 6 months and 10.47 (SD 20.94) at 12 months. The MDC was 4.72 at 3 months, 5.20 at 6 months and 4.71 at 12 months.

**Conclusions:**

The calculated MCID in patients following surgery for unstable ankle fractures ranges from 10.5 to 15.0 at 3–6-month follow-up and from 7.5 to 11.4 at 6–12-month follow-up, depending on moment and method.

## Clinical relevance

This study is clinically relevant as OMAS results should be interpreted with known MCID and MDC.

## What is known about this subject?

Previous studies show the MCID of the OMAS with a single method and not every study used multiple measurement moments.

## What this study adds to existing knowledge?

This study calculates the MCID of the OMAS using multiple methods and multiple measurement moments. Therefore, this study results in multiple MCID values and future studies can use the most suitable MCID for the interpretation of the OMAS scores.

Level of evidence: I (diagnostic test).

## Introduction

The Olerud Molander Ankle Score (OMAS) is a widely used validated Patient Reported Outcome Measure (PROM) in clinical research regarding ankle injuries since its introduction in 1984 [1–4]. The aim of a PROM is to take the patients’ perspective of health, illness and benefits of health interventions into consideration in a reliable, valid and feasible way [5]. It supplements clinical knowledge about the effectiveness of interventions, which is based on physiological measures [6]. The best measurement of treatment quality should include the patients’ opinion of the outcomes [7]. In addition, scoring a patients’ outcome may improve patient care by adding functional insight to clinical trials.

The OMAS is a scoring system designed specifically for patients following a fracture of the ankle, with a scale from 0 (severe disability) to 100 (no disability) [4]. Nine parameters are used, including pain, stiffness, swelling and various functional abilities, as shown in Table 4 of the appendix. Several studies have shown that the OMAS is a reliable and valid outcome score in various different languages, although it has not been validated in Dutch yet [8–12].

To interpret the effects of a certain treatment, it is essential to take the Minimal Clinically Important Difference (MCID) into consideration. The MCID is defined as the smallest difference in PROM scores which the patient considers beneficial [13, 14]. For clinical research, it is important to determine this threshold. If the mean change score is lower than the MCID, there is no clinical benefit of the treatment, even if the change is statistically significant.

The objective of this study was to determine the MCID of the OMAS using anchor-based and distribution-based methods for patients undergoing open reduction and internal fixation of unstable ankle fractures with syndesmotic injury at 3, 6 and 12-month follow-up.

## Materials and methods

This study was a prospective cohort study, conducted alongside a randomized controlled trial. To define the MCID of the OMAS, we extracted data from the RODEO trial, an international randomized controlled trial on removal of syndesmotic screws after ankle surgery, which included patients between January 2017 and April 2019 [15]. Patients were randomized to either routine removal of the syndesmotic screw or removal on demand. The primary outcome of this study was the functional outcome at 12 months using the OMAS. Patients in the on demand group underwent removal of syndesmotic screw(s) in case of symptomatic implants like pain, causing restricted range-of-motion, explicit request of the patient, or infection necessitating removal. Patients in the control group underwent routine removal of the syndesmotic screw(s) within 8–12 weeks following the index procedure.

For this cohort, patients were excluded if they did not complete the questionnaire at two consecutive moments or did not complete the anchor questions. The Medical Ethical Review Committee granted approval for the RODEO trial including this parallel study to determine the MCID based on the data of this clinical trial (METC AMC 2016-197, NL58539.018.16).

The Dutch version of the OMAS was completed at 3, 6 and 12-month follow-up and at baseline, patients were asked to make an estimate of pre-trauma function.

Because the pre-trauma function was an estimation of the OMAS, this measurement has been excluded from MCID calculations.

To be able to assess the MCID of the OMAS, anchor questions were added at 6 and 12 months as described by Walenkamp et al. [16]. These anchor questions were on a global rating of change scale (GRC) from − 5 (much worse) to + 5 (much better).

### Statistical analysis

There are several ways to define the MCID of a PROM. These methods can be divided into anchor-based and distribution-based methods [13, 17]. The first approach uses external anchors as a benchmark to determine the MCID. These can be both objective anchors like Range of Motion (ROM), or patient-reported anchor questions. With patient-reported anchor questions, patients determine themselves if they experienced a clinical benefit of the treatment.

The first anchor-based method is the mean change, which was first described by Jaeschke et al. [14].

Our anchor question had a range from − 5 to 5 in which categorized scores − 5 to − 3 as ‘worsened’, − 2 to − 1 as ‘minimal worsening’, 0 as ‘unchanged’, 1 and 2 as ‘minimal improvement’ and 3–5 as ‘improved’ [18].

Within these groups, the mean change in OMAS score was conducted with confidence intervals as: $\mathrm{Meanchange }\pm 1.96 \left(\frac{\mathrm{SDchange}}{\sqrt{n}}\right)$. The mean change in the ‘minimal improvement’ group was considered the MCID [6].

The second anchor-based method is the received operation characteristics (ROC) curve analysis.

Patients were divided into two groups, according to their answers of the anchor questions at 6 and 12 months. These categories were 0 (unchanged); and 1–5 (improvement), which excluded the patients that reported themselves as worsened [18]. Subsequently, the distribution of the change on the OMAS scores could be plotted in both groups. This results in the Youden index for every cut-off value, which is a performance indicator of the MCID and the highest index found is the optimal cut-off point [19]. As a next step, specificity and sensitivity for each cut-off in the change score were calculated. For every cut-off, the sensitivity was plotted against 1-specificity, which resulted in a ROC curve. The upper left corner on the ROC is, therefore, the MCID. This point accounts for the score with the least amount of misclassification.

The distribution-based method was also used to determine the MCID of the OMAS. The MCID was calculated by multiplying the SD of the score on 3, 6 and 12 months by 0.5. In the context of comparing group averages, an effect size of 0.2 is considered small, 0.5 is considered medium and 0.8 as a large effect size and, therefore, 0.5 SD was chosen [20].

The second distribution-based method is the minimal detectable change (MDC), which describes the minimal amount of a patient's measure that exceeds the measurement error. This MDC is closely related to the modified reliable change index (RCI) [21]. By calculating the statistical characteristics of the sample and take the significant changes into account, the MDC is compared to the probability that the change has occurred by chance [17]. This MDC can be calculated from the standard error of the measurement (SEM) using $1.96* \sqrt{2}*\mathrm{SEM}$

Following testing for normality, the Kruskal–Wallis test was used to test for significance among the different anchor groups, which would implicate that the categories are sufficiently discriminative. Correlation was calculated using Spearman’s rho test to determine effectiveness of the anchor questions. Correlation coefficients were interpreted as very high positive correlation (0.9–1.0); high positive correlation (0.7–0.9); moderate positive correlation (0.5–0.7), low positive correlation (0.3–0.5); and negligible correlation (0.0–0.3) [22].

Statistical significance was set at a *p* value of 0.05 or less. Normal distribution was assessed with histograms and boxplots. All analyses were performed using SPSS version 26 (IBM, Armonk, New York, NY).

## Results

One hundred and eighty-three patients filled in the OMAS at some point in time during the RODEO trial.

A total of 148 patients were available in the study after the exclusion of 35 patients, because these patients did not fill in the OMAS at 2 consecutive moments or because of missing anchor questions.

At 3 months, 119 patients completed the OMAS. At 6 months, 127 patients completed the OMAS. At 12 months, 132 patients completed the OMAS.

Mean age was 47 years, and 94 patients (63.5%) were male. Baseline characteristics are provided in Table 1.

**Table 1:** Baseline characteristics

Variable	
Age in years, mean (SD)	47 (19–76; SD 14.8)
Men, *n* (%)	94 (63.5%)
BMI, mean (SD)	27.9 (5.1)
Active smoker, *n* (%)	32 (21.6%)
Diabetes Mellitus, *n* (%)	4 (2.7%)
Injury type, *n* (%)	
Weber B	33 (22.3%)
Weber C	76 (51.4%)
Maissoneuve	35 (23.6%)
Isolated syndesmosis injury	2 (1.4%)
Missing, *n* (%)	2 (1.4%)
Removal syndesmotic screw(s), *n* (%)	
Routine removal	70 (47.3%)
On demand removal	78 (52.7%)
Of which had the syndesmotic screw(s) removed < 12 months, *n* (%)	24 (30.8%)
Complications, *n* (%)	16 (10.8%)
Wound infections, *n* (%)	8 (5.4%)

The median OMAS improved significantly from 3 months to follow-up at 12 months (*p* < 0.001). At 3 months, the median OMAS was 55.0 (IQR35.0–70.0). This increased to 70.0 (IQR 50.0–85.0), at 6 months and to 85.0 (IQR 60.0–95.0) at 12 months.

Table 2 includes an overview of the MCID for each method and measurement moment.

**Table 2:** MCID for every used method at different moments in the follow-up

Method				
*Anchor-based methods*	*3–6 months* *MCID*		*6–12 months* *MCID*	
Mean Change	15.0		9.5	
ROC curve (AUC)	12.5 (0.72)		7.5 (0.78)	
*Distribution-based methods*	*3 months* *MCID*	*6 months* *MCID*		*12 months* *MCID*
0.5 SD	10.5	11.4		10.5
MDC	4.7	5.2		4.7

### Anchor-based methods

With the anchor-based method, the mean change within the groups with ‘worsened’, ‘minimal worsening’, ‘unchanged’, ‘minimal improvement’ and ‘improvement’ between the different measurement moments is displayed in Table 3.

**Table 3:** Mean change in OMAS based on anchor group

	3–6 months, mean (SD, 95% CI)				
	Worsened	Minimal worsening	Unchanged	Minimal improvement	Improvement
Mean change	7.5 (10.6, − 87.8 to 102.8)	− 1,25 (11.1, − 18.9 to 16.4)	3.1 (15.8, − 10.1 to 16.3)	15.0 (17.5, 9.4–20.6)	19.6 (16.9, 15.1–24.0)

	6–12 months, mean (SD, 95% CI)				
	Worsened	Minimal worsening	Unchanged	Minimal improvement	Improvement
Mean change	NA	− 4.3 (10.5, − 10.4–1.8)	4.4 (10.5, − 4.4 to 13.2)	9.5 (17.1, 3.1–15.9)	14.0 (14.1, 10.3–17.7)

These categories discriminated significantly with *p* = 0.002 from 3 to 6 months and *p* < 0.001 from 6 to 12 months.

There was a positive correlation according to the correlation coefficient between the change in OMAS and the five anchor groups in both the change from 3 to 6 months (0.291, *p* = 0.002) and 6 to 12 months (0.374, *p* < 0.001). This confirms the adequacy of the GRC categories.

**Fig. 1:**
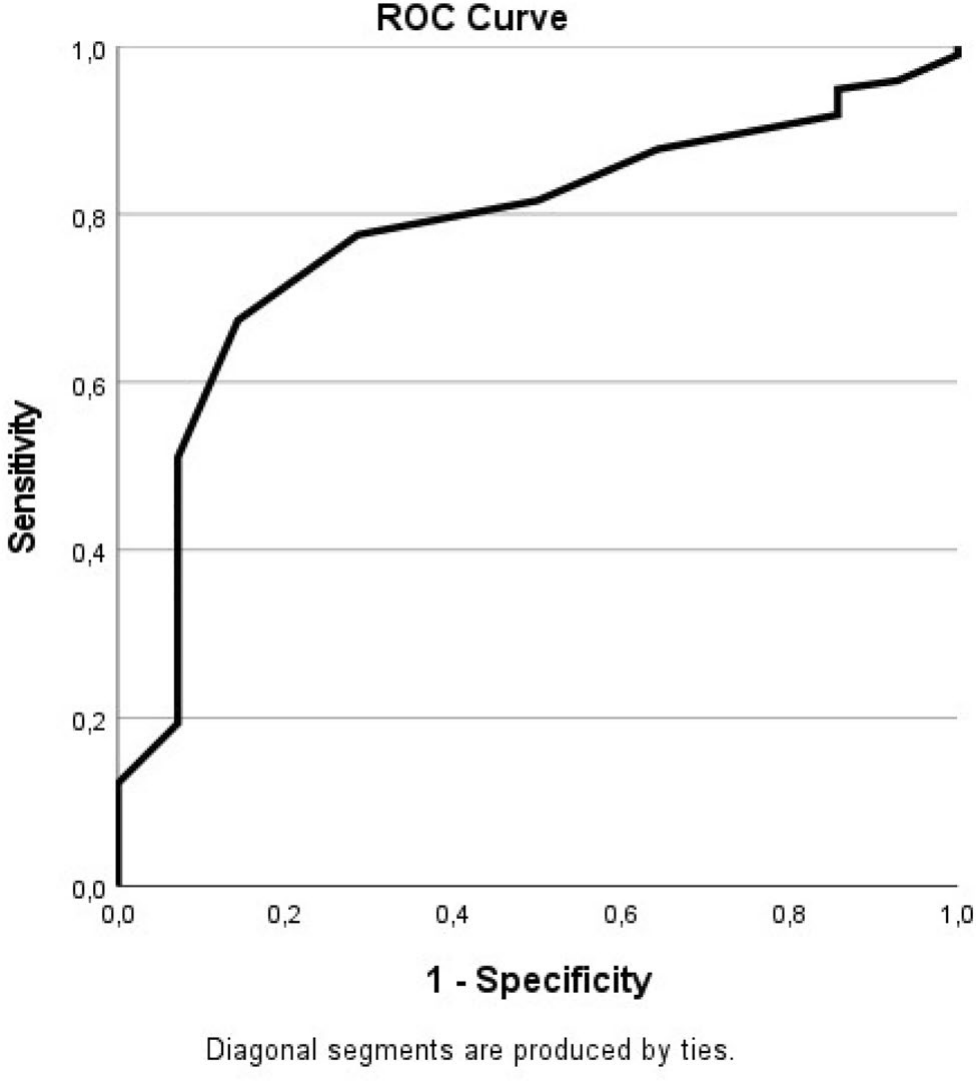
ROC Curve of the difference between 3 and 6 months. The ROC Curve showing correlating sensitivity and specificity of the OMAS as a measurement instrument. MCID 12.5, Youden index 0.53, Sensitivity 0.67, Specificity 0.86, AUC 0.72

**Fig. 2:**
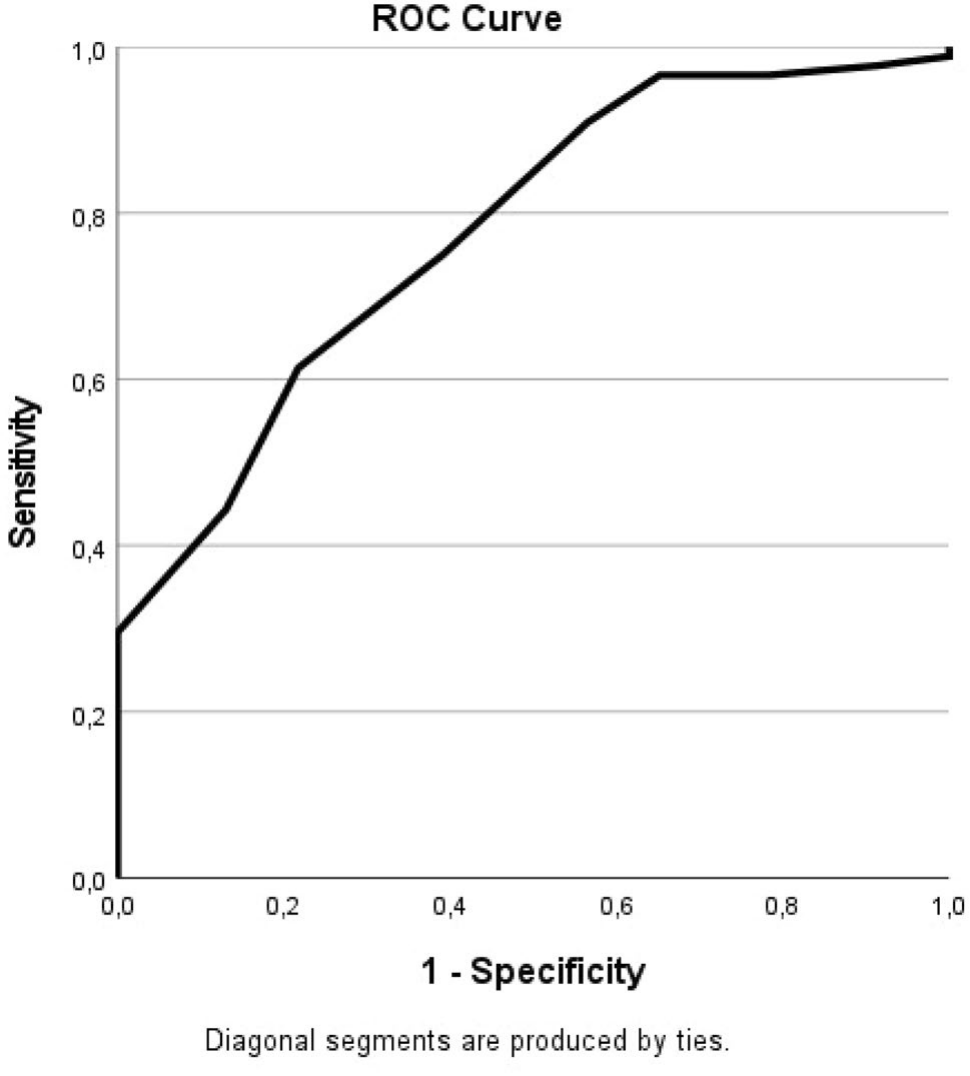
ROC Curve of the difference between 6 and 12 months. The ROC Curve showing correlating sensitivity and specificity of the OMAS as a measurement instrument. MCID 7.5, Youden index 0.40, Sensitivity 0.61, Specificity 0.78, AUC 0.78

The second anchor-based method was the ROC curve, which was used on both intervals. The ROC curves for both intervals are shown in Figs. 1 and 2.

For the difference in OMAS between 3 and 6 months, the MCID was 12.5, the Youden index was 0.53 with a sensitivity of 0.67 and a specificity of 0.86. The area under the curve (AUC) was 0.72.

For the difference in OMAS between 6 and 12 months, the MCID was 7.5, the Youden index was 0.40 with a sensitivity of 0.61 and a specificity of 0.78. The area under the curve (AUC) was 0.78.

### Distribution-based methods

The distribution-based method using half a standard deviation (0.5 SD) produced a SD for every measurement moment and subsequently three MCIDs using 0.5 SD values. At 3 months, the MCID was 10.5 (SD 21.0). At 6 months, the MCID was 11.4 (SD 22.7). At 12 months, the MCID was 10.5 (SD 20.9).

The second distribution-based method was the minimal detectable change (MDC). With this method, the MDC for the different measurement moments was 4.7 with a SEM of 1.7 at 3 months, 5.2 with a SEM of 1.9 at 6 months and 4.7 with a SEM of 1.7 at 12 months.

## Discussion

We used four different methods to calculate the MCID for the OMAS in patients who underwent surgical stabilization of unstable ankle fractures with syndesmotic screw placement. The different methods and measurement moments resulted in a MCID ranging from 7.5 to 15.0.

Other studies that reported the MCID for the OMAS were by Gausden et al. in 2018 and McKeown et al. in 2021 [10, 23]. The study by Gausden was a prospective trial over a 3-year period, with follow-up moments at 3, 6 and 12 months. A total of 132 patients were included. However, only 15% of patients filled out the PROMs at all three FU moments and 39% at two different follow-up moments. Compared to this study, our cohort had a higher percentage of responses. The MCID of Gausden et al. was calculated using a distribution-based method (0.5 SD) based on study population parameters at their 12-month time point and no anchor-based method was used. An overall MCID of 11.5 was found, with 17.6 points difference when comparing the 3–6-month follow-up, and 3.8 when comparing the 6–12-month follow-up. The main weakness of using this method is that it uses a standard formula for calculating the MCID rather than the gold standard of improved (or decreased) function viewed from a patients’ perspective. The main purpose of a PROM is to measure outcome from the patient’s perspective and therefore, we suggest that this method is less suitable for calculating the MCID [6]. Gausden et al. showed that there is a larger difference in MCID at different time points with a larger MCID at the 3 versus 6 months interval compared to the 6 versus 12 months interval (MCID of 17.6 versus 3.8, respectively) [23]. Our results also show a larger MCID at 3 versus 6 months, compared to 6 versus 12 months interval, which could be explained by the fact that the functional improvement following surgery is larger between 3 and 6 months than between 6 and 12 months [15, 24, 25].

In a more recent study, McKeown et al. collected data from 620 patients with an ankle fracture at 10 and 16 weeks [10]. The MCID in this study was calculated using anchor questions. A MCID of 9.7 points was found in this study. The main limitation of this study is the short period of follow-up. Functional outcome following the open reduction and internal fixation of an ankle fracture increases over time. Egol et al. found a significant increase in ankle function when comparing the 6 months and the 1-year follow-up, while Sanders et al. even documented continued improvement in ankle fractures up to 24 months following injury using the OMAS [25, 26]. Therefore, the MCID calculated by McKeown et al. has low external validity, and is only applicable in studies on short-term outcomes [10]. Using the correct MCID when designing a study is of importance, because a low MCID value may result in overestimating the beneficial effect of a treatment, whereas a high MCID value may falsely lead to concluding that a treatment has failed, while in fact, patients benefitted from the treatment [17]. Therefore, the use of multiple follow-up moments in our study is of value.

To determine the MCID, anchor-based methods are preferred, as they include a definition of what is minimally important. The most common used anchor-based method is the receiver operator characteristic (ROC) curve analysis [17, 27]. The main downside of the anchor-method is that is does not take the measurement error of a PROM into account and it is, therefore, of value to use both the anchor and the distribution method. Our ROC curve resulted in an AUC of 0.72 and 0.78 for the intervals of 3- 6 months and 6–12 months, respectively. This is an acceptable accuracy of the test and therefore of the MCID of the OMAS [28, 29].

When using the distribution method, one of the methods actually calculates the minimal clinical detectable change rather than the minimal clinical important change. The MDC measures whether a change is truly a change or merely a result of standard variation within the test. Therefore, it may reflect a true change in outcome score but it does not necessary mean that this change it also clinically relevant. However, it is important to know the MDC as the MCID should always be higher than the MDC. If this is not the case, a test would not be sensitive enough to measure clinically relevant changes.

There are several limitations when interpreting the results of the present study. First of all, the patients in this study all underwent open reduction internal fixation (ORIF) of an unstable ankle fracture. These fractures tend to have a worse outcome compared to more stable fractures, which do not need operative fixation which could have influenced the MCID [26, 30, 31]. Furthermore, the Dutch version of the OMAS has not been validated yet, which may affect the measuring qualities of the questionnaire. Lastly, there was quite a high percentage lost to follow-up. This is a common phenomenon in orthopedic trauma studies but it could have influenced the results of this study.

This study is relevant for future studies and for the application of results in a clinical setting. We would advise to take the MCID into account that matches the follow-up moment as the OMAS changes per measurement moment and additionally, this should at least exceed the MDC (4.7–5.2). Furthermore, it is of value that our study used multiple methods including anchor-based methods to compare different methods and to compare the different MCID values because the MCID decreases over time. There is not enough evidence to advice one specific method and accordingly one overall MCID, which could be the topic of future studies.

Additionally, this study used data from a randomized controlled trial (RCT) and, therefore, uses high-quality data.

## Conclusion

The calculated MCID in patients following surgery for unstable ankle fractures ranges from 10.5 to 15.0 at 3–6-month follow-up and from 7.5 to 11.4 at 6–12-month follow-up, depending on moment and method.

## Data Availability

All data are published in this manuscript, the cited manuscripts or the supplementary appendix. Data can be provided upon request and in agreement of terms. No individual participant data were used.

## Appendix 1

See Table

**Table 4:** Olerud Molander ankle score

Parameter		Degree	Score
I	Pain	None While walking on uneven surface While walking on even surface outdoors While walking indoors Constant and severe	25 20 10 5 0
II	Stiffness	None Stiffness	10 0
III	Swelling	None Only evenings Constant	10 5 0
IV	Stair-climbing	No problems Impaired Impossible	10 5 0
V	Running	Possible Impossible	5 0
VI	Jumping	Possible Impossible	5 0
VII	Squatting	No problems Impossible	5 0
VIII	Supports	None Taping, wrapping Stick or crutch	10 5 0
IX	Work, activities of daily life	Same as before injury Loss of tempo Change to a simpler job/part-time work Severely impaired work capacity	20 15 10 0

^4^.
